# A Review of Existing Studies Reporting the Negative Effects of Alcohol Access and Positive Effects of Alcohol Control Policies on Interpersonal Violence

**DOI:** 10.3389/fpubh.2015.00253

**Published:** 2015-11-16

**Authors:** Jessica L. Fitterer, Trisalyn A. Nelson, Timothy Stockwell

**Affiliations:** ^1^Spatial Pattern Analysis and Research Laboratory, Department of Geography, University of Victoria, Victoria, BC, Canada; ^2^Centre for Addictions Research of British Columbia, Psychology Department, University of Victoria, Victoria, BC, Canada

**Keywords:** violence, alcohol policy, alcohol price, alcohol trading hours, alcohol outlet density

## Abstract

Alcohol consumption often leads to elevated rates of violence yet alcohol access policies continue to relax across the globe. Our review establishes the extent alcohol policy can moderate violent crime through alcohol availability restrictions. Results were informed from comprehensive selection of peer-reviewed journals from 1950 to October 2015. Our search identified 87 relevant studies on alcohol access and violence conducted across 12 countries. Seventeen studies included quasi-control design, and 23 conducted intervention analysis. Seventy-one (82%) reported a significant relationship between alcohol access and violent offenses. Alcohol outlet studies reported the greatest percentage of significant results (93%), with trading hours (63%), and alcohol price following (58%). Results from baseline studies indicated the effectiveness of increasing the price of commonly consumed alcohol, restricting the hours of alcohol trading, and limiting the number of alcohol outlets per region to prevent violent offenses. Unclear are the effects of tax reductions, restriction of on-premises re-entry, and different outlet types on violent crime. Further, the generalization of statistics over broad areas and the low number of control/intervention studies poses some concern for confounding or correlated effects on study results, and amount of information for local-level prevention of interpersonal violence. Future studies should focus on gathering longitudinal data, validating models, limiting crime data to peak drinking days and times, and wherever possible collecting the joint distribution between violent crime, intoxication, and place. A greater uptake of local-level analysis will benefit studies comparing the influence of multiple alcohol establishment types by relating the location of a crime to establishment proximity. Despite, some uncertainties particular studies showed that even modest policy changes, such as 1% increases in alcohol price, 1 h changes to closing times, and limiting establishment densities to <25 outlets per postal code substantively reduce violent crime.

## Introduction

Alcohol access and consumption has contributed to escalated levels of violence including domestic (1–4), sexual (5), and interpersonal assaults (6–9). Consistent effects are represented by a comprehensive evaluation of 563 injury cases from 16 different countries showing that intoxicated patients had a higher likelihood of a violence-related injury than any other cause (10). Generally, the risk of interpersonal violence increases with the frequency and volume of alcohol consumption (11–13), and in relation to certain types of drinking environments and activities (6, 14–21). For instance, consuming alcohol in a restaurant is unlikely to lead to alcohol-related violence (22, 23), whereas intoxicated patrons inside and around bars have created spatial clusters of violent offenses in these areas (6, 24).

The relationship between alcohol availability and violence is complex, including an individual’s biochemical, psychological, and social responses to alcohol consumption and their environment. Researchers have established that intoxication ignites violent behavior in those predisposed to aggression (25) and theorize that consumption leads to weakened inhibitions [see Ref. (26)] and relaxed normative behavior (i.e., perceived allowance of aggression) causing the increased risk of alcohol-related violence inside and around drinking premises (27). Considering place-based theories, it is also likely that alcohol serving establishments attract perpetrators of violent crime by grouping targets for victimization, ultimately leading to clusters of crime in regions with greater alcohol availability [e.g., routine activity theory (27, 28)]. For these reasons, policies that manage alcohol consumption and interaction of intoxicated persons are paramount for public safety.

Relaxing the mechanisms that control the availability of alcohol is likely to increase violent offenses by yielding higher consumption and promoting intoxicated high-risk patrons to interact (29). Increases in violence have been linked with decreased alcohol prices (30–32), extended trading hours (6, 33), and increased alcohol outlets densities (34–36) including both on (e.g., taverns, hotels, bars, pubs, and clubs) and off (e.g., retail stores, off sales) retailers; though, alcohol availability continues to rise in many regions worldwide (29, 34, 35, 37–39). The trend in policy relaxation indicates a need to synthesize the effects of alcohol access on violent offenses to inform those tasked with improving public health and safety.

The goal of our review was to summarize the effects of alcohol policy on violent offenses through restrictions on alcohol availability. We extend the findings of existing alcohol policy reviews. First, by evaluating the effectiveness of price, trading hours, and alcohol outlet density on violent crime simultaneously, where previous reviews have focused on one (32, 33) or two (34) alcohol access influences. Second, we update the syntheses of alcohol policy effects, which have seen a considerable increase in publication since 2010 rising from one to five publications per year between 1995 and 2010 to ~12 a year afterward. Using a health geography perspective, we provide a brief critique on the quality of study designs, data (sources and aggregation), geographic scale, and methods. We suggest ways to improve evidence-based information for decision making, and highlight areas of uncertainty for future policy studies to address.

## Methods

### Study Selections

Using the web of science and Google scholar, we performed a comprehensive search of the available literature using a combination of key terms integrated in the following Boolean query: “([(alcohol tax^⋆^ OR alcohol cost^⋆^ OR alcohol price^⋆^ OR alcohol outlet^⋆^ OR alcohol outlet density^⋆^ OR alcohol trading hours OR alcohol sales OR alcohol availability OR licensing OR bars^⋆^ OR pubs^⋆^ OR hotels^⋆^ OR on-premises OR off-premises) AND (violence OR assault^⋆^ OR domestic violence OR homicide OR interpersonal violence OR rape)]).” We searched available studies from January 1950 to October 2015 resulting in 888 selections, which we refined to 798 after excluding reviews, editorial materials, and meeting abstracts to focus on primary research. From the 798 studies, we excluded studies reporting the effects of alcohol access policy on crimes other than violence and studies that associated alcohol consumption/access other than price, trading hours, or outlet outlets to violent crime/injuries. Our final selection of 87 studies included papers that analyzed a change or cross-sectional assessment of the effects of alcohol tax, price, hours of trading, or establishment density on violent injury or crime data.

### Study Synthesis

Studies were summarized by author, date of publication, place, and year/s of study. Outcome variable, exposure variables, study design, control data, and key findings were recorded. Any statistical concerns were noted. To demonstrate the quality of information available for alcohol price, trading hour, and outlet density policy decision making, we quantified the proportions of intervention (i.e., quasi-control studies), panel, time-series, and cross-section datasets. We considered the range of countries represented by analysis and if data distributions were mapped over time and space to facilitate the interpretation of data and results for policy makers, health, and police personnel. We also considered the frequency of researchers using control groups or the independent effects of demographics, socio-economics, and concurrent polices on violent occurrences.

To evaluate study quality, and addressed gaps in analysis techniques, we summarized data sources and modeling methods. For the outcome variable, we considered the source of information and if the violent event data were directly alcohol attributable. To assess overall power of the statistical analyses, we considered how data (outcome and exposure) were aggregated over analysis units (e.g., census tract versus block groups) and time. We indicated prominent statistical models and if spatial or temporal dependence was tested or modeled to control for biased reductions in SEs (40). We monitored if studies using short time periods (e.g., consumption in previous months) or small geographic units (e.g., alcohol outlets in neighboring communities) included lagged temporal (e.g., alcohol consumption in previous time period) or spatial effects (e.g., number of alcohol outlets in neighboring regions) when estimating violent occurrences.

We synthesized alcohol policy findings by dataset structure. Categories included: cross-section (data collected or aggregated over one time period, representing multiple individuals or regions), time-series (individual or aggregated data collected from one region over time), panel (aggregated data collected over multiple regions and time-periods), or intervention (data collected before and after an alcohol policy change, either in a time-series or panel analysis) categories. Studies using control group or intervention data were evaluated first. Second, the contributions of time-series and cross-section studies were incorporated as policy-based evidence. To represent trends across datasets, we calculated the percentage reporting a significant relationship between alcohol availability and violence within each dataset category.

## Results

### Study Selections

Eighty-seven studies met the inclusion criteria, representing analyses of 12 price/tax, 19 trading hour, and 56 outlet density alcohol policy effects on violent crimes/injuries. The majority of studies were conducted in the United States (60%) and Australia (17%). For a complete list of countries see Table 1. Twenty-one of the 70 geospatial studies included maps. Ten authors represented their study area, 14 mapped violent offense rates/counts, 10 depicted the spatial distribution of alcohol outlets, and two mapped their coefficient results (7–9, 41–58).

**Table 1:** **Count of publications by country of analyses**.

	Country											
	United States	Australia	England/Wales	Sweden	Brazil	Norway	New Zealand	Denmark	Finland	Scotland	Canada	Colombia
Alcohol price or tax	7	0	2	1	0	0	0	1	1	0	0	0
Alcohol trading hours	1	6	4	2	2	1	0	0	0	1	1	1
Alcohol outlet density	44	9	0	0	0	1	2	0	0	0	0	0
Total	52	15	6	3	2	2	2	1	1	1	1	1

### Study Designs

Of the 87 studies reviewed, 17 [(alcohol price (*n* = 1), alcohol trading hours (*n* = 12), and alcohol outlet density (*n* = 4)] employed quasi-control or comparative crime statistics in their analysis. Control group data consisted of comparative region’s crime statistics [alcohol price (*n* = 1), trading hours (*n* = 9), and outlet density (*n* = 2) (41, 42, 52, 59–65)], establishments not adopting an alcohol policy change (6), non-alcohol-related crime statistics (66), alcohol outlets (67), and random locations (56, 57). Twenty-three studies conducted intervention analysis where crime data were analyzed pre and post a change in alcohol price/tax (*n* = 4, 33%), alcohol trading hours (*n* = 16, 84%), or alcohol licenses (*n* = 3, 5%). Proportionately cross-sectional datasets were the dominant across the literature (53%), with panel (18%), intervention time-series (16%), intervention panel (10%), and time-series (2%) following. Alcohol price/tax policy changes were most often assessed using panel datasets (50%), alcohol trading hours by intervention time-series assessments (76%), and alcohol outlet density studies by cross-sectional datasets (77%).

### Violent Crime Data

The sources of violent crime information and types of crimes studied are summarized in Tables 2 and 3. Police reports (*n* = 61, 70%) and assaults (*n* = 33, 38%) were the prominent data source and violent event type. Two alcohol price studies used crime data flagged as alcohol attributable (68, 69). Thirteen alcohol trading hour studies stratified crime data to primary drinking days per hour. Other trading hour studies related violent crime data to alcohol by collecting criminal event data in and around establishments (18, 19) or relating crime and consumption data through survey information. Whereas, three of the 56 alcohol density studies restricted crime data to peak drinking hours (8, 56, 70) or weekend offenses (70) to infer alcohol-attributable offenses. While others used alcohol-attributable crime from police (57) or conducted analysis of crime around alcohol outlets (54, 56, 57).

**Table 2:** **Source of violent crime statistics**.

	Source of crime information				
	Police reports	Hospital admission records	Survey	State records	Health statistics
Alcohol price or tax	3	2	4	1	2
Alcohol trading hours	15	4	0	0	0
Alcohol outlet density	43	9	4	0	0
Total	61	15	8	1	2

**Table 3:** **Types of violent crimes/injuries studied**.

	Violent crime/injury type					
	Assaults	Aggregated violent crime	Domestic violence	Homicides	Child abuse	Injury caused by violent offense
Alcohol price or tax	5	0	1	3	1	2
Alcohol trading hours	11	3	2	3	0	0
Alcohol outlet density	17	26	8	2	2	1
Total	33	29	11	8	3	3

### Methodologies

Overviewing the 70 spatial studies, we determined that a variety of delineated units were used to represent how violent crime rates were related to alcohol access. Census tracts (*n* = 17) and zip/postal codes (*n* = 10) were the most commonly applied geographic units; for a full list, see Table 4. A large proportion of the total 87 studies (*n* = 78) used regression modeling techniques to analyze the extent to which alcohol availability is associated with violent crime (price (*n* = 12), trading hour (*n* = 14), and alcohol density (*n* = 54). Of these studies, 74 included controls for concurrent policy changes, area, and/or individual characteristics to recognize independent effects, exclusive of alcohol, on violent offense rates. Specifically, 19 considered the concurrent alcohol consumption characteristics, alcohol availability laws, and changes in police force densities on the occurrence of violent incidences.

**Table 4:** **Studies categorized by applied spatial units**.

Spatial unit	Studies	Count
Census tracts	(4, 47, 49–51, 53, 58, 71–80)	17
Zip/postal codes	(1, 2, 7–9, 23, 44, 81–83)	10
States	(69, 84–89)	7
Block groups	(45, 55, 90–93)	6
Census blocks	(43, 52, 94–96)	5
Cities	(63, 97–99)	4
Point level	(54, 56, 57)	3
Municipalities	(61, 100, 101)	3
Countries	(41, 42)	2
Economic regions	(68, 102)	2
Local government areas	(48, 103)	2
Police-defined areas	(104,105)	2
Neighborhoods	(46, 106)	2
Rural communities	(70)	1
Metropolitan areas	(107)	1
Counties	(5)	1
Buffered college areas	(108)	1
Electoral wards	(67)	1
Total geospatial studies		70

The majority of alcohol price studies (*n* = 10, 83%) employed linear regression modeling. In the case of an intervention analysis, autoregressive integrated moving average (ARIMA) (*n* = 2) or linear models with a dummy variable were used to understand the effects of alcohol price changes on violent crime rates (84, 107, 109, 110). Alcohol trading hours studies (*n* = 19) most often employed interrupted time-series analysis either through linear regression (*n* = 4), generalized linear models (*n* = 5), or ARIMA models (*n* = 5). To a lesser extent, descriptive analysis (62), distribution comparisons (111, 112), time-series structural model (113) or spatial lag regression model (67) were employed. Poisson and Negative Binomial regression models were the dominant methods of estimating the effect of regional alcohol outlet density (*n* = 18, 30%) on interpersonal violence, only two studies used methods other than regression to study the association between alcohol access and violent offenses (43, 54).

To ensure data independence between spatial units, one of the eight cross-sectional alcohol price studies accounted for unit dependence (69), and 32 of the 56 alcohol outlet density studies controlled or tested for lag 1 (first-order contiguity) autocorrelations, or specified spatially lagged dependency effects between analysis units. One intervention panel (42) and four intervention time-series studies (6, 60, 65, 113) tested for serial temporal autocorrelation or difference time-series to stationarity, while two intervention panel (61, 84), one panel (85), and one time-series study (66) explored temporally lagged effects including alcohol consumption or alcohol policy laws on the occurrence of reported violence.

### Policy Results

#### Alcohol Price and Violent Crime

Of the 12 alcohol price or tax policy studies, seven (58%) reported significant policy effects on violent crime and injuries (68, 69, 84, 86–88, 102). One of the four policy intervention studies reported a decrease in assaults and robberies following a 1991 increase in alcohol taxes (84). Of the six panel studies, four documented a significant relationship between the price of beer and violent event, finding that increases in price had the ability to reduce violent injury, assault, and the probability of being assaulted (68, 69, 86, 102). Similarly, cross-sectional studies reported that a 1% in state level excise beer tax was associated with a 0.33% reduction in child abuse rates (87) and 3.10–3.50% reduction in domestic abuse cases (88). The synthesis was less clear for tax reductions, indicating no significant change in violent crime across Nordic countries (107, 109, 110) and no significant association between alcohol United States tax variances and homicides (85, 89).

#### Alcohol Trading Hours and Violent Offenses

Out of the 19 alcohol trading hour studies, twelve reported significant policy effects on violent crime rates (63%). Seven of the eleven intervention analyses, using control data (6, 59, 61–64, 66) and four of the six intervention studies, without control data, found trading hours to significantly affect violent crime (18, 111, 113, 114), particularly trading hour extensions leading to increases violent crime. Cross-sectional analysis also found that countries with longer trading hours (up to 1 h) had four or more violent or gun-related crimes per 100,000 persons per year (5). Contrasting significant findings, controlled intervention studies reported no significant effects on the rates of hospital-reported/police-reported assaults and aggregated violence categories when restricting patrons to re-enter on-premises establishments (60), allowing variance in on-premises closing times (i.e., staggered closing) (65, 67), and opening off-premises outlets on Saturday opening (41, 42). Panel studies also found no significant change in assaults after a 1-h extension in alcohol sales (97) and the implementation of staggered closing times for on-premises drinking establishments (112).

#### Alcohol Outlet Density and Violent Offenses

Among the 56 studies of alcohol outlet density selected, 52 represented significant outcomes. Most notably, intervention analysis indicated that the number of assaults significantly reduced after outlet licensing surrenders in Los Angles (50, 71) and a 3.2% reduction in on-premises licenses in Buckhead, United States lead to a twice greater decrease in the level of violent crime (~6%) (52). All 11 panel studies (1, 2, 7, 44, 50, 51, 54, 71, 81, 98, 104) indicated an increasing trend between alcohol outlet density and the occurrence of violence. Longitudinal analysis also showed that additional outlets increased the number of violent street crimes by one-three events per block group over 3 years in Norfolk Virginia (90). A positive and significant correlation between alcohol outlet density changes and violent crimes (115) including homicides (98) were found across the time-series studies covering 22 and 35 years of data. The results of panel and time-series studies were consistently reflected by the 43 cross-section studies offering 39 significant positive associations between alcohol density and violent crimes (3, 8, 9, 23, 43, 45–49, 53, 55–58, 70, 72–80, 82, 90–96, 99, 103, 105, 106, 108, 116). Only four studies reported inconclusive results (insignificant coefficients) when relating domestic abuse (4, 100), child abuse (83) and assault (101) incidences to alcohol outlet densities across municipalities, zip codes, and census tracts.

## Discussion

### Policy Synthesis

Our literature search identified 87 relevant studies on alcohol access and violent offenses conducted across 12 countries, though the majority of analysis was completed in the United States (*n* = 52). Seventy-one studies (82%) reported a significant relationship between alcohol access and violent offenses. Alcohol outlet studies represented the greatest percentage of significant results (93%), with trading hours (63%), and alcohol price following (58%). Relationships between alcohol policy and violent offenses were reported across all study designs including policy intervention studies using control data to cross-sectional overviews (Table 5).

**Table 5:** **Summary results of the selected publications**.

	Percent of significant/substantive findings				
	Intervention time series	Intervention panel	Panel	Cross-section	Time-series
Alcohol price or tax	0%	50%	67%	100%	N/A
Alcohol trading hours	66%	25%	100%	100%	100%
Alcohol outlet density	N/A	100%	100%	91%	100%
Number significant	8	5	14	42	2

*Presented are the percent of studies reporting significant/substantive policy effects on violent injury/crime categorized by study design, policy type, and combined policy types*.

Consistent trends emerged across the alcohol availability polices. Using cross-section, panel, and intervention research designs, with various levels of data aggregation, researchers identified that alcohol tax and price increases significantly reduce violent offenses including child abuse, intimate partner violence, assaults, and injuries (68, 69, 86–88, 102). Particularly, when price increases were directed toward commonly consumed alcohol, such as beer. Effects were independent of regional and individual’s socio-economic and demographic characteristics and consistent when monitoring the change in the number of emergency room attendees for alcohol-related violence (68).

Overviewing both intervention and time-series studies results, substantive trading hour restrictions (e.g., 24 h access changed to regulated closing hours or more than 2 h restriction in on-premises alcohol sales hours) led to marked reductions in homicides, battery, domestic violence, and assaults (59, 61, 62, 64, 113, 114). At on-premises locations, staggered closing times reduced regional rates of violent offenses by 34% (111) and restricting re-entry reduced 50% of offenses occurring on-premises (18). The effects of trading hour restrictions were also consistent for alcohol-attributable assault injuries (111). Asymmetrically, the extension of trading hours, up to 1 h, increased assaults (6, 61, 63) and homicide rates (66).

Consistently intervention, time-series, panel and cross-section studies found that increases in spatial density of alcohol outlets led to higher rates violent crimes, with the effects magnified in marginalized communities (46, 47, 49, 53, 55, 70–72, 74–76, 80, 90, 91, 93, 94, 99, 103, 106, 108, 116). A strong positive and significant association between on-premises license densities and assaults were observed by multiple researchers (9, 23, 48), with local-level analysis clearly showing clusters of assaults, violent crime, and violent crime flagged as alcohol-attributable [e.g., Ref. (57)] around alcohol outlets (54, 56, 57, 90, 92). Exponential increases in violent crime were observed in postal code regions with >25 establishments (3, 8). Separating outlet effects by licensing type, numerous studies found higher amount of violent offenses around a greater amount of bars or on-premises licenses (excluding restaurants) (1, 9, 23, 45, 52–54, 56, 58, 93) with the effects of bars doubling violent offenses in economically deprived areas (23). In rural settings, the same increase in regional violent offenses rates was seen with higher densities of off-premises outlets (103). A greater density of off-premises licenses were also related to a greater amount of gunshot wounds (53) and intentional injuries (96) in regions of the United States and Australia.

Generally, increased access led to higher rates of violent crime with drinking establishments acting as hot spots of violent crime, though a smaller percentage of studies (18%) reported insignificant effects [alcohol price (42%), alcohol trading hours (37%), and alcohol outlet density (7%)]. Researchers relating homicides to variances in state-wide alcohol tax rates were unable to identify significant associations (85, 89). The power of analysis was questioned by the low variability in tax rates across states by the authors (89), and we caution against relating state-wide policy to individual crime events, though intervention or individual data are needed to properly assess the effects of alcohol tax on heinous crimes.

We also found asymmetry between the influences of tax increases and reductions. Tax increases (84, 87) led to significant reductions in violent crimes while tax reductions had no significant effects on violent crime in other regions (107, 109, 110). Possibly population characteristics (Nordic countries versus United States) or differences in methodologies (panel versus intervention) were dictating the asymmetric pattern, though further analysis is needed to report the general effect of tax reductions on violent crime, particularly considering how tax affects real price indexing for consumers.

A few trading hour policies reported no significant reduction in violent crime (97), namely after the opening of off-premises locations on Saturdays (41, 42), restricting patron re-entry (60), and staggering closing times of on-premises locations (65, 67, 112). It is likely that the marginal effects on crime were caused by people planning for regulated closures of off-premises outlets (41, 42). And while modest re-entry restrictions and staggered closings did not create substantive reductions crime rates in some regions, the peaks in the spatial and temporal patterns of alcohol-attributable crime were effected (18, 59, 65, 97, 112). Policies that dictate the spatio-temporal pattern of crime are essential for proactive policing, though a larger number of local studies are needed to document consistency of the effect.

In terms of alcohol outlets, a minority of studies reported insignificant effects (4, 83, 100, 101). Some uncertainty remains regarding the effects of on- versus off-premises alcohol establishments on domestic violence [e.g., Ref. (1, 104)]. Spatial scale of alcohol access measurement (municipalities/zip codes) and consumption data [e.g., Ref. (4)] may have masked effects of outlets on violent crime though a greater amount of data collected on “ the place of intoxication” is needed to make conclusive results regarding domestic violence and the types of alcohol outlets. Preliminary studies indicate that individuals drinking in pubs, taverns, hotels, and bars increased the likelihood of domestic violence (82) and child maltreatment (73); however, alcohol consumption rather than place may be influencing domestic altercations. Survey-respondent data are needed to confirm results.

### Study Design Considerations

Overall, consistent policy trends were identified among a vast heterogeneity of study designs, outcome measures, and statistical models. Unfortunately, an insufficient amount of intervention (*n* = 23) or control (e.g., comparative region or point crime statistics) (*n* = 15) studies exist. Twenty-six percent of the studies explored the change in violent crime post an alcohol policy intervention, the remaining 74% conducted panel, time-series, or cross-section assessments studying trends in the variances of alcohol access and crime. There are ways to tease out causality in a plethora of observation, mostly ecological (aggregated unit) studies. For example, a greater number of researchers could consider the concurrent implications of independent alcohol polices and active policing on the occurrences of violent crime or reporting. Currently, only 32% of studies have considered the simultaneous effects of alcohol policies (3, 4, 48, 70, 77, 78, 84, 87, 88, 112) including quota abolishment on alcohol sales (41, 109, 110), outlet densities (5, 86–88), dry laws (86, 87), or changes in police force capacity (6, 61, 70, 86, 114); however, these independent factors play a pivotal role in the estimation of violent crime.

To enhance the reliability of estimation effects garnered from cross-sectional or panel studies with limited longitudinal data, future studies could implement a cross-validation or “hold back” method of model validation where a proportion of the dataset is used to build the alcohol availability–violence model and a portion of data are withheld to use as validation for model predictions. Currently, only two studies validate the efficiency and transferability of their model using test data (79, 98). It is equally important to address statistical assumptions of regression models prominently used across the literature. We found that across the geospatial studies (*n* = 70) 23 tested for spatial dependence and four (6, 42, 60, 65, 113) of the time-series studies tested for temporal serial autocorrelations. Untested datasets are vulnerable to autocorrelation which can result in clustered residuals and artificial decreases in the SEs (117, 118).

We caution against studies attributing survey respondent crime information to geographic measurements of alcohol access (e.g., state-wide alcohol taxes or postal code level outlet densities) to infer causation. Using ecological characteristics to understand behaviors of individuals can lead to fallacies of inference from unmatched scales (119) as does generalizing individual results to a group. Studies that use generalized measures of alcohol polices without control groups, or intervention analysis, are vulnerable to falsely attributing violent crime rates to alcohol access policy. Therefore, to limit the potential biases, study designs, whenever possible, should compare response and predictor data between comparable scales, over time, or subsequent to a policy change. Results should be compared between study designs and across scales when synthesizing information from study designs attributing individual responses to environmental factors in the individual’s area.

In terms of data, there are opportunities to record alcohol use indicators and place of last consumption to develop joint distributions between crime and alcohol use, though the majority (*n* = 59) of crime datasets were not linked to intoxication or place of consumption. We recognize that confining studies to alcohol-attributable data would present a limited scope with primary data sources including surveys (*n* = 8), and generalized police reports (*n* = 61). However, hospital admissions data collection (*n* = 15) presents the opportunities to collect consumption information (i.e., blood alcohol level) and wherever possible, we suggest studies use crime data collected at detailed spatial or temporal scales to strengthen causality. These may include geo-located crimes in or around alcohol establishments, crimes occurring during on-premises closing times, or crimes recorded between peak drinking hours [see Ref. (120)]. Authors have recognized the benefits of stratifying crime reports to strengthen model or analysis results (8, 18, 19, 56, 57, 65, 67, 70). Such that, crimes occurring outside of drinking hours, that may have a different spatial or temporal distribution, do not mask the relationship between alcohol access policies and violent crime especially in the absence of intervention analysis where it is plausible to attribute changes in the occurrence of crime to the change in alcohol policy.

Considering exploratory data, a substantial portion (68%, *n* = 38) of the alcohol density studies assessed the relative impact of on-premises versus off-premises outlets, 18 studies analyzed the aggregated effects of alcohol outlet densities reducing the usefulness for setting outlet density restrictions by type. We suggest studies continue to undertake comparative analysis between types of outlet establishments and seek to collect sales data in which, for instance, establishments of different size and capacity are not measured equally in the model. Currently, the results between on- and off-premises alcohol outlet density and violent outcomes measures are variable for some violence types and standardization of outlet grouping and sensitivity analyses on the collinearity between outlet density types is essential to consolidate results.

### Geographic Perspective for Future Research

The majority of alcohol availability studies used a spatial unit (e.g., states, cities, census tracts, neighborhoods, and blocks) to associate the count or rates of offenses against regionally specific socio-demographic and alcohol policy variances, such as changes in price (68, 84, 107), hours of closing (5, 61), or alcohol outlet densities (43, 79, 90, 91). Census tracts and zip code areas were the dominant analysis units (39%), with few studies conducted at units smaller than a census tract (43, 45, 46, 52, 54–57, 90–96, 106). As a result, available information for evidence-based policy making is focused on expected effects of broad scale (e.g., state wide or city wide) policy changes to alcohol tax, trading hours, or outlet densities. More information from local-level or event-level analysis is needed to understand if targeting alcohol availability restrictions toward problem venues or specific neighborhoods would have the same or greater net effect on violent crime reduction. For example, establishment licensing decisions are often made considering neighboring crime rates and outlets within 500 m radius, though very few results are presented at matching scales (54, 56, 57). We recognize that standardizing crime rates becomes particularly hard at the local level when persons move between analysis units (e.g., blocks). However, using crowd sourced population estimates from social media (121) or remotely sensed data of night-time lights (122) can offer new ways of quantifying dynamic (changing) populations’ estimates at smaller units than the most common postal codes or census tracts. For example, researchers can use geo-located (i.e., *x* and *y* coordinates) social media status updates (e.g., tweets), searched using open source software, such as twitteR (https://cran.r-project.org/web/packages/twitteR/index.html), as a proxy for the spatio-temporal location of sub populations and their sentiments [e.g., Ref. (121)]. Twitter data has proven to improve the prediction of various crime types (123). It is also possible to redistributed population estimates from larger census units using indicators of land use (night-time) lights and other attributes (e.g., land slope) to estimate where the residential population spends the majority of their time on the landscape (e.g., LandScan data), creating population estimates as fine as 1 km spatial resolution (122).

Many researchers have also focused on reporting results as an effect size, such that a unit increase in alcohol price, hours of trading, or rate of establishments creates a percentage increase in violent offenses across study areas. While vital for policy-based evidence, the spatial interpretation of the alcohol–violence relationship is lost. Understanding where populations are most vulnerable to alcohol access is useful for local policy making, such as choosing restrictions on alcohol outlet locations, targeting trading hour restrictions to specific problem areas, or implementing minimum prices at problem venues. Mapping is shown to aid in monitoring the statistical assumption such as residual patterns (124), interpreting, and communicating policy results (29, 37) though only 21 of the 70 spatial analyses included maps (7–9, 41–51) of which two were limited to a depiction of study area (41, 42). Therefore, opportunities for enhancing spatial representation are available.

With the size of analysis units decreasing with the advent of Geographical Information Systems opportunities are presented to study the spatially lagged effects of alcohol availability on neighboring crime rates or apply spatial modeling techniques (Geographically Weighted Regression) to address heterogeneity of alcohol–crime relationships over board spatial areas [e.g., Ref. (58)]. Currently, 25 (2, 7–9, 23, 43–45, 55, 69–72, 74, 76, 80, 82, 90, 91, 93, 94, 96, 103, 104, 116) studies incorporated spatial lagged effects of which the majority considered the effects of alcohol access in neighboring units, though it is possible to study multiple lagged effects to understand the distance decay or alcohol accessibility changes on violent occurrences to create evidence-based policies for establishment hours and densities. For example, Grubesic and Pridemore (43) used spatial cluster detection and autocorrelation analysis to identify where, at what density, and to what extent assaults were clustering around different types of establishments and three other point analysis studies (54, 56, 57) used multiple analysis buffers around individual establishments to indicate the extent at which crime clusters around particular outlets. However, more studies applying smaller spatial units should consider distance effects to make conclusive recommendations for the allowable proximity of establishments or density of establishments in neighboring units.

Using geo-located event crime data, we can use point pattern analysis to study relationships between alcohol and crime. Currently, almost all (97%) of the alcohol policy studies reviewed have conducted a spatial analysis or modeling using contiguous analysis units. Point patterns assessments can provide information on how crime clusters around establishments (56, 57), during various drinking hours (54), and pricing schemes for preventative planning at specific sites. Recent publications using point data and density mapping have been shown to provide great insight into the influences of different drinking environments (125) though to capitalize on these methods future analysis needs to shift from regression-based modeling to spatio-temporal point pattern analysis perspective [see Ref. (126)].

## Conclusion

Repeatedly findings showed that targeting the price of commonly consumed alcohol (e.g., beer), restricting the days of sales, and limiting the clustering of alcohol establishments can have protective effects for violence perpetration. Summarizing 87 studies, representing 12 alcohol price changes, 19 trading hour per day modifications, and 56 alcohol outlet density studies, we found a lack of control or intervention study designs leaving policy personnel to rely heavily on the results from cross-sectional analyses. What remains unclear are the effects of tax reductions, the effectiveness of moderate trading policies (lock-out), and the varying effects of establishment types on violence. We believe cross-section studies could improve in quality by holding data back to conduct validation tests on their models, more commonly consider the concurrent effects of alcohol polices and law enforcement on crime reporting, and conduct analysis across smaller spatial units providing visualization of crime and alcohol access over time and space for decision making. In terms of data, researchers should capitalize on opportunities to collect the joint distribution of crime occurrences and intoxication in plausible cases such as during hospital admissions. Further, we believe that there is a greater opportunity to access the variable effect of establishment types on crime by disaggregating licensing types and control for any collinearity between density measurements within the analysis. Despite methodological limitations and some variable findings, it is essential to remember that well designed studies have indicated that even modest policy changes including a 1% increases in alcohol price, 1 h changes in closing times, or limitations on establishment densities to <25 outlets can lead to substantive reductions in violent crime/injury occurrences. Recognizing the diversity of datasets, levels of aggregation, and methods the majority of studies indicated that increasing the price, limiting the hours of sales, and restricting the number of establishments are effective policies for alcohol-attributable violent injury/crime management.

## Author Contributions

TS designed the concept of work, JF conducted study acquisition and analysis, and all authors contributed to the interpretation of results and contributions. TS, JF, and TN revised the manuscript for intellectual content and approved the work for publication.

## Conflict of Interest Statement

The authors declare that the research was conducted in the absence of any commercial or financial relationships that could be construed as a potential conflict of interest.
